# Learning to Rank Figures within a Biomedical Article

**DOI:** 10.1371/journal.pone.0061567

**Published:** 2014-03-13

**Authors:** Feifan Liu, Hong Yu

**Affiliations:** 1 Department of Computer Science, University of Wisconsin-Milwaukee, Milwaukee, Wisconsin, United States of America; 2 Department of Quantitative Health Sciences, University of Massachusetts Medical School, Worcester, Massachusetts, United States of America; 3 VA Central Western Massachusetts, Northampton, Massachusetts, United States of America; University of Warwick, United Kingdom

## Abstract

Hundreds of millions of figures are available in biomedical literature, representing important biomedical experimental evidence. This ever-increasing sheer volume has made it difficult for scientists to effectively and accurately access figures of their interest, the process of which is crucial for validating research facts and for formulating or testing novel research hypotheses. Current figure search applications can't fully meet this challenge as the “bag of figures” assumption doesn't take into account the relationship among figures. In our previous study, hundreds of biomedical researchers have annotated articles in which they serve as corresponding authors. They ranked each figure in their paper based on a figure's importance at their discretion, referred to as “figure ranking”. Using this collection of annotated data, we investigated computational approaches to automatically rank figures. We exploited and extended the state-of-the-art listwise learning-to-rank algorithms and developed a new supervised-learning model BioFigRank.

The cross-validation results show that BioFigRank yielded the best performance compared with other state-of-the-art computational models, and the greedy feature selection can further boost the ranking performance significantly. Furthermore, we carry out the evaluation by comparing BioFigRank with three-level competitive domain-specific human experts: (1) First Author, (2) Non-Author-In-Domain-Expert who is not the author nor co-author of an article but who works in the same field of the corresponding author of the article, and (3) Non-Author-Out-Domain-Expert who is not the author nor co-author of an article and who may or may not work in the same field of the corresponding author of an article. Our results show that BioFigRank outperforms Non-Author-Out-Domain-Expert and performs as well as Non-Author-In-Domain-Expert. Although BioFigRank underperforms First Author, since most biomedical researchers are either in- or out-domain-experts for an article, we conclude that BioFigRank represents an artificial intelligence system that offers expert-level intelligence to help biomedical researchers to navigate increasingly proliferated big data efficiently.

## Introduction

There are 1.2 zettabytes (10^21^) of electronic data being generated each year and a federal effort in U.S. has recently been kicked off aiming to manipulate and mine the massive amounts of information more efficiently [1]. In the biology domain, descriptive data is also getting richer and richer. For example, hundreds of gigabytes of DNA and RNA sequencing data can be generated in a week for less than US$5,000 [2]. As such tackling and understanding the big data has become a demanding challenge confronting virtually all fields of biology [3], many technologies, such as data integration, cloud and heterogeneous computing [2], [4], [5] and software engineering [6], have been developed to make sense of the big data [7], [8]. In this study, we focus on developing computational approaches to tackle the big data challenge posed by millions of biomedical figures.

Figures published in biomedical literature are typically experimental evidence for knowledge discovery and biomedical researchers need to search for figures to validate facts and to formulate and test novel research hypotheses. It is estimated that over 100 million figures have been published [9].

The importance for searching and mining those figures has motivated vigorous research in this area. The Subcellular Location Image Finder (SLIF) system [10] is the first system that targets figures in biomedical literature. SLIF extracts and analyzes the fluorescence microscope figures to capture sub-cellular location. Rafkind et al. [11] and Shatkay et al. [12] developed computational approaches to automatically classify biomedical figures into types (e.g., gel and microscopy image). The BioText search engine [13] and Yale Image Search [14] have been developed allowing researchers to search for biomedical figures.

However, nearly all research in figure search [10], [13], [14], embraces a “bag of figures” assumption, and the retrieval figures are ranked mainly by query-based relevancy without considering the relationship among figures within the same article. The “bag of figures” approach does not distinguish figures from each other. Some figures may carry more important roles such as representing main findings in the articles while other figures may play a supportive role to main figures. A biologist who is looking for a biological fact may be more interested in an article in which the fact is not only supported with experimental evidence (figure) but also judged as the central topic of the article.

The new figure search system we are developing Biomedical Figure Search (http://www.figuresearch.org) [9], [11], [15]–[18] has taken different directions: we identify the semantic relations between figures and their associated text. Our evaluation results have shown that associated texts are important for figure comprehension [15]. On the other hand, texts associated with a figure are typically redundant [19], we therefore developed *FigSum* to automatically generate a structured text summary for each figure [17]. We also associate figures to texts appearing in the abstract of the article [9], [20].

**Figure 1:**
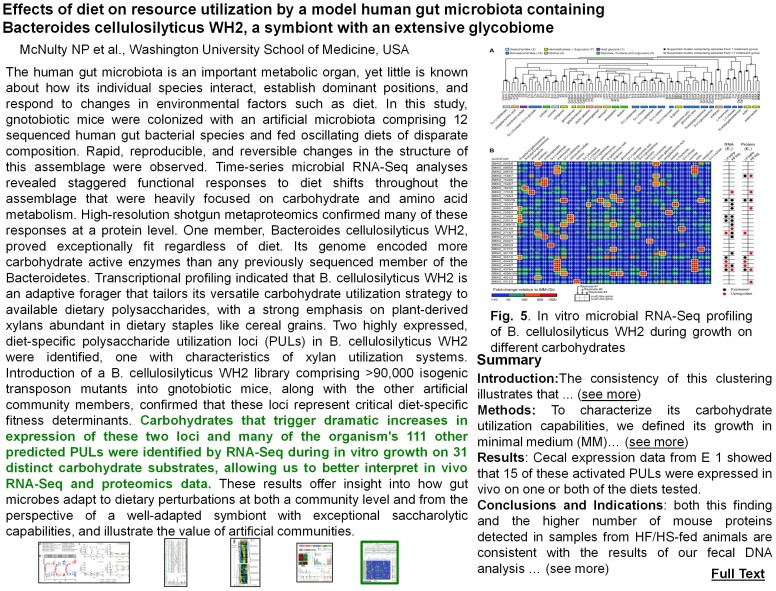
Illustration of the figure-text-summary user interface (Illustrative content is taken from the article DOI: 10.1371/journal.pbio.1001637).

The figure-text-summary user-interface, as shown in Figure 1 that was published in our previous work [16], enables biomedical researchers to efficiently browse through the main content without the access of the PDF-format full-text article. Figures appearing in a full-text article can be ranked by their importance (from the authors' point of view) [16], therefore this succinct user-interface, integrated with figure ranking, can provide a one-page summary of an average of ∼30 pages of full-text articles. The 30-foldinformation reduction offers an effective solution to address the big data challenge researchers face today. Figure ranking (FR) can also be integrated to improve information retrieval and extraction.

We have previously developed a preliminary unsupervised approach for figure ranking [16]. In this study, we develop innovative supervised machine-learning “learning-to-rank” approaches for figure ranking. Our contributions are:

1. We are the first group to develop supervised machine-learning approaches for ranking figures in a biomedical article.
2. We explore domain-specific features as well as linguistic-motivated features for the listwise “learning-to-rank” method.
3. We implement and evaluate a new loss function for listwise learning using the top two permutation probability distributions.
4. We conduct an extensively comparative study on the figure ranking task, competing machine computation versus human intelligence as well as benchmarking different computational models.

### Related Work

Ranking is one of the most important tasks of relation learning. In the field of natural language processing (NLP), many algorithms have been developed and successfully applied to different applications, such as speech recognition [21], [22], information extraction [23], [24], information retrieval [25], [26], question answering [27], syntactic parsing [28]–[30], and machine translation [31], [32].

In the machine learning community, the so-called “learning-to-rank” methods have been successfully applied to information retrieval (IR) tasks, which include three categories: pointwise [e.g., [33]], pairwise [e.g., [34]], and listwise approaches [e.g., [35]].

The pointwise approach [33] casts ranking problem into regression or classification on single objects. The pairwise approach transforms ranking into classification on object pairs into two categories (correctly ranked and incorrectly ranked), and the use of Support Vector Machines (SVM), Boosting, and Neural Network as the classification model lead to the methods of RankSVM [34], RankBoost [36] and RankNet [37]. The advantage of these two approaches is that existing theories and algorithms on regression or classification can be directly applied. However, there are also problems. First, neither of them model the ranking problem directly, so the objective of learning is formalized as minimizing errors in classification or regression, rather than minimizing errors in ranking itself. Second, the number of candidate objects associated with each individual set (e.g., each query in IR task) varies largely, which will result in training a model biased toward individual set with more candidate objects.

The listwise approach overcomes the drawbacks of the pointwise and pairwise approaches by tackling the ranking problem directly. There are two branches in listwise ranking: directly optimizing the evaluation metrics [38]–[40] and minimizing a listwise loss function [35], [41], [42]. The study in this paper falls into the second branch and is closely related to the work of Cao et al. [35], who proposed one of the first listwise methods, called ListNet. They defined the loss function using the cross entropy between two probability distributions of permutations; one is from the predicted ranking and the other is from the ground truth. ListNet uses gradient descent algorithm to train a linear neural network model. Similarly, ListMLE [41] also used a neural network model for training, yet to minimize the likelihood loss function. Another listwise approach, RankCosine [42], defined a cosine loss function between two score vectors; one is from the predicted scores and the other is from the ground truth. Instead of using neural network, RankCosine chose to employ a generalized additive model for the learning. In this study, we make a first attempt to investigate supervised learning method on the figure ranking task by incorporating different types of features into a listwise learning-to-rank framework.

Although prior studies have explored different learning algorithms on various ranking tasks, figure ranking within biomedical articles has not yet been studied. As we have stated earlier, existing figure search systems [10], [13], [14], [43], [44] and other image-related tasks, including ImageCLEF — the evaluation competition of cross language image retrieval as part of the Cross Language Evaluation Forum (CLEF) [45], are all based on the “bag of figures” assumption without considering the relations between figures within an article. We have previously reported an unsupervised approach for figure ranking [16]. In this work, we significantly extend the previous study by exploring supervised machine learning.

## Materials and Methods

### Exploring Listwise Learning Approach for Figure Ranking

ListNet is a representative listwise learning-to-rank model. A detailed model description is reported in [35]. Here, we describe how listwise learning can infer the ranking preferences among figures within a biomedical article.

In figure ranking, a ListNet instance is a list of figures in each article, and each figure is represented by a vector consisting of features (details of features are described in the later sections). The objective of learning is formalized as minimization of a listwise loss function defined using the distance between two permutation probability distributions derived from the gold standard ranking and system predicted ranking respectively. More formally, we denote the ranking function as *f_w_* that is based on the neural network model (*w*) in ListNet. Given a figure feature vector *x^(i)^_j_* (*i* indicates the index of biomedical articles, *j* indicates the index of figures in an article) *f_w_* assigns a score to it. For the list of figure vectors *x^(i)^* we obtain a list of scores *z^(i)^* = (*f_w_*(*x^(i)^*_1_), … , *f_w_*(*x^(i)^_n(i)_*)) where *n^(i)^* is the number of figures in the *i*th article. Therefore, the total loss with respect to the training data is:
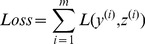
(1)where L is a listwise loss function, *y^(i)^* is the corresponding list of scores by human (here we use the reciprocal of the rank assigned to each figure as its ground truth score), and *m* is the number of articles in the training data.

For a specific loss function, ListNet transforms two lists of scores (*z^(i)^ and y^(i)^*) into probability distributions based on top one probability model. For instance, based on a list of scores (*z^(i)^*) from the ranking function, the probability of the *j*th figure's being ranked on the top (for simplicity, we ignored the article index *i* hereafter):
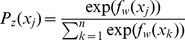
(2)


With cross entropy as metric, the loss function L in Eq. (1) becomes:
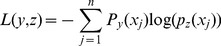
(3)where *P_y_*(*x_j_*) is the probability of the *j*th figure's being ranked on the top based on ranking scores assigned by human. Then, the gradient of the loss function with respect to the neural network model parameter *w* can be calculated as:
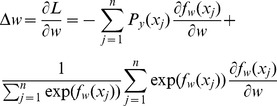
(4)Eq. (3) is used in gradient descent learning, where the *w* is updated with *w* = *w* - *r*×Δ*w* (*r* is the learning rate) in each iteration.

When we applied ListNet on figure ranking, we also used a simple adaptive method to dynamically change the learning rate *r* by multiplying a constant (0.875), if it remains above a threshold (10^−6^) and the evaluation metric on the training data improves or remains the same in the current iteration.

### BioFigRank: Extending Listwise Loss Function Beyond Top One Probability

As discussed earlier, the loss function of ListNet [35] is defined based on the distance between two permutation probability distributions derived from the gold standard ranking and system predicted ranking. Due to the larger numbers of candidates to be ranked in the traditional IR tasks, the current loss function in ListNet is limited to using only top one permutation probability distribution. We hypothesize that moving beyond top one probability distribution better characterize ranking information from both human annotation and automatic prediction, resulting in an improved loss function which may enhance the learning ability for parameter optimization. For traditional information retrieval tasks, it is impractical to go beyond top one probability distribution due to the expensive computation caused by the larger number of candidates to be ranked. But for the figure ranking task, the number of figures per biomedical articles is relatively smaller (the average is around 5 [9]) which offers perfect opportunities to explore the effectiveness of extending the loss function beyond top one probability distribution. In this study, we developed BioFigRank, which implemented a new loss function based on top 2 permutation probability distribution, to automatically rank figures within a biological article.

Based on the theoretical definition of top *k* probability in [35], top 2 probability on subgroup *g*_2_(*x*_1_,*x*_2_) can be derived by:
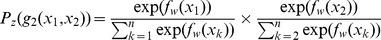
(5)where the subgroup *g*_2_(*x*_1_,*x*_2_) contains all the permutations in which the top 2 figures are exactly (*x*_1_,*x*_2_); thus, top 2 probabilities form a probability distribution over collection *G*_2_, which consists of n*(*n*-1) subgroups (e.g. *g*_2_(*x*_1_,*x*_2_)).

We replace the top one probability in Eq. (3) with top 2 probability, and the loss function becomes:
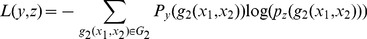
(6)The new gradient of the loss function based on top 2 probabilities is derived below:
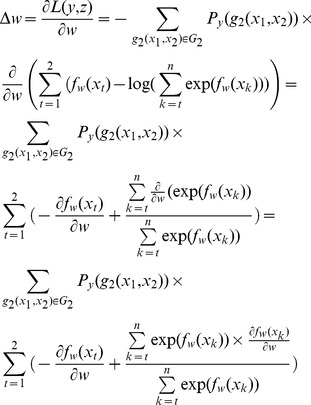
(7)


### Learning Features for Figure Ranking

We represent each figure with its caption text and associated text, from which we extract word features. We follow our previous work [16] to extract associated text, which comprises of words in sentences that mention the figure, as well as the preceding and following two sentences. Since our previous studies have concluded that biomedical articles can be typically represented by four rhetorical categories: introduction, methods, results, and discussion (IMRAD) [e.g., [9], [15], [16]] and we speculate that such categorization may be useful for figure ranking as important figures may be more likely described in the result and discussion section. We therefore added the IMRAD based features. All the features we explored can be grouped into four categories, containing 89 features as follows.

#### Centrality features

We speculate that the more important a figure is, the more it is the center topic of an article. In our previous work [16], we found such centrality features improved figure ranking. Specifically, we evaluated six cosine similarity features between figures and articles, where each figure was represented by its associate text or caption and each article was represented by its title, abstract and full-text. In this study, we extended the centrality features to include figure's degree of centrality in the article, including the cosine similarities between the figure (represented using caption, associated context and caption plus associated context respectively) and each IMRAD section (i.e., introduction, methods, results, and discussion) of the article, and the similarities between the figure (represented using caption plus associated context) and the article as a whole (represented using title, abstract, full text, respectively).

#### Frequency features

The more frequently a figure is discussed, the more likely that the figure is important. We therefore added the frequencies of a figure appearing in each individual IMRAD section, as well as the weighted frequencies in results and discussion section where the weight for each paragraph is obtained based on the cosine similarity between the paragraph and the article's title or abstract. We also normalized the frequency by its section length and added the normalized value as additional features.

#### Topic based features

We employed latent Dirichlet allocation (LDA) analysis [16] to identify the latent semantic topics by taking each paragraph in each article as an individual document. It is assumed that each paragraph is generated from multiple implicit topics and words in each paragraph are generated based on two multinomial distributions: distribution over topics for each paragraph, and distribution over words for each topic. Two variations on topic representation were explored: one using words with higher probability in the topic; the other using paragraphs belonging to the topic.

Once we identified the topics, we calculated the cosine similarity between each topic and the corresponding article's title and abstract. Based on the similarity score, we selected the top 4 topics with higher scores to examine their relationships with each figure. Specifically, we calculated the cosine similarity scores between each figure (represented as caption, associated context, and caption plus associated context) and each of the top 4 topics with two different representations, respectively (word or paragraph) as features.

#### Structural features

In addition to relations between the figure and the article, we also examined relations among figures as well as figure specific internal structural features as follows:

- Position feature: the numerical position that a figure appears in an article. For instance, the value for the first figure is 1.
- Link feature: We hypothesize that the stronger the association between a figure and other figures, the more importance of the figure. We first calculated cosine similarity between each figure pair (using their caption plus associated context), and for each figure we chose the mean and stand deviation with regard to its similarity scores with others as features.
- Sub-figure feature: Biomedical figures frequently contain sub-figures. We speculate that there is an association between the number of sub-figures and the importance of a figure. To capture the number of sub-figures, we applied regular expression-based pattern matching on figure captions (for example, “([a-fA- F])[\.|\,|\:]+” can recognize sub-captions like “a.”, “A:” or “c,”).

### Forward Greedy Feature Selection (FGFS)

Feature selection is important for any machine-learning-based classification tasks. For conventional classification tasks, feature selection can be performed by investigating the correlation or dependency between class labels and individual features, such as information gain and chi-square methods. However, those approaches cannot be directly applied on the figure ranking task in the listwise learning-to-rank framework, because there is no explicit class label associated with each instance (i.e. a set of figures in each article). In this study, we explored the forward greedy feature selection(FGFS) approach, which have been shown effective in automatic keyword extraction [46].

**Figure 2:**
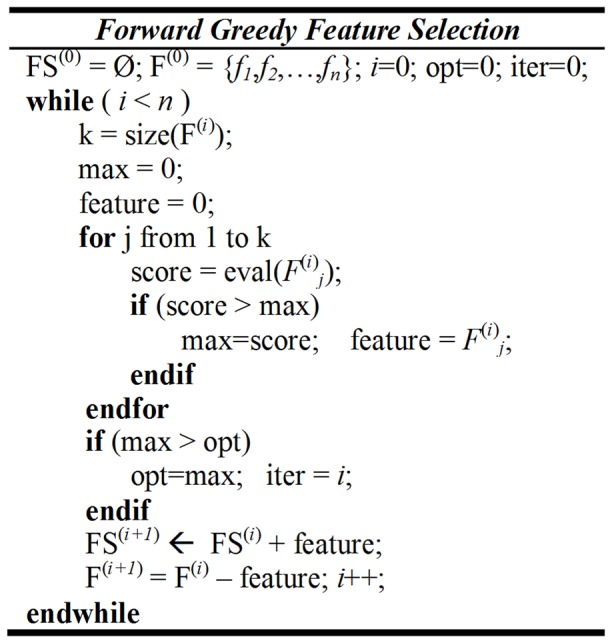
Forward greedy feature selection algorithm.

As shown in Figure 2, forward feature selection starts with an empty feature set and at each iteration, adds one feature that has achieved the best performance gain. The output is the best performance score (*opt*), corresponding iteration index (*iter*) and selected feature set (FS^(*iter*)^).

### Data and Evaluation Metrics

**Figure 3:**
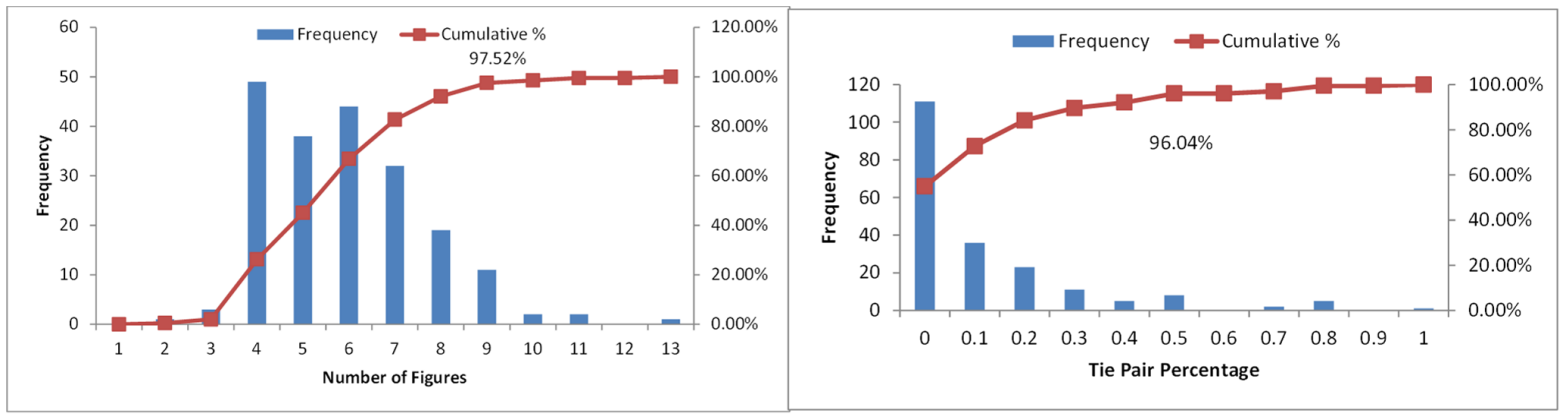
Statistics on the gold standard data. *A*(left): Distribution of articles with different numbers of figures in an article; *B*(right): Distribution of articles with different percentages of tie pair relations in an article.

The annotated data is a collection of 202 biomedical articles from three journals (*Journal of Biological Chemistry*, *Proceedings of the National Academy of Sciences* and *PLoS Biology*) [16]. The average number of figures per article is 5.9±1.75. Figure 3A shows that most articles in this dataset contain 4–6 figures and 97.52% of the articles have 9 or less figures.

The corresponding authors manually ranked the figures in their publication. A tie relation represents two or more figures that are judged by the authors as equally important. We calculated tie pair percentages for each article, which is the number of figure pairs that were annotated equally important divided by the total possible figure pairs contained in that article. For example, assuming one article has three figures, the annotation is “Fig 2, Fig 1 = Fig 3” (“ = ” indicates the tie relation), then the tie pair percentage would be 1/3. As shown in Figure 3B, in the 202 annotated articles, 45% of them have at least one tie relation. The tie pair percentage of most articles (96.04% of them) is 0.5 or less, and only few articles were annotated with a high percentage of tie relations. This collection of annotated figure-ranking data is used as our gold standard dataset.

We used three metrics for the evaluation. First, we used a new mean weighted error rate (WER-RK), which takes into account the reference rank information associated with wrongly recognized figure pairs. This modified metrics has shown advantages in [16] over the original mean weighted error rate proposed by [16] to measure the errors of recognizing pair relations. Specifically, WER-RK assigns more penalties on wrongly ranked pairs if they contain more important figure, and its value on each article can be calculated by:
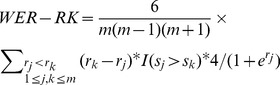
(8)where *m* is the number of figures in the article, *s_j_* and *s_k_* are the system ranks of figure *j* and *k*, respectively, *r_j_* and *r_k_* are the reference ranks of figure *j* and *k*, respectively, I(.)is the indicative function.

Another metric we used is normalized discount cumulative gain (NDCG), a widely used evaluation criteria in document retrieval and in the learning-to-rank community [e.g., [37]]. The NDCG value on figures in each article can be obtained by:
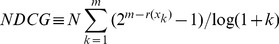
(9)where *k* is the predicted rank for each figure, *m* is the number of figures in the article, *r*(*x_k_*) is the reference rank of the *k*th ranked figure in the system prediction, *N* is chosen so that a perfect ordering receives NDCG score 1.

Finally, we used a weighted error rate on the first rank (WER-FR) to evaluate the identification of the most important figure in each article, which is weighted by the deviation distance of the system rank on the most important figure assigned by human from the reference rank (i.e., 1) as follows.
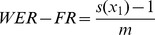
(10)where *s*(*x*_1_) is the system rank on the figure *x*_1_ that is ranked on the top by human, *m* is the number of figures in the article.

We present our results as ***∼***WER-RK (“1-WER-RK”), NDCG and ***∼***WER-FR (“1-WER-FR”) so that larger values indicate better ranking performance.

### Models Implemented on Figure Ranking for Performance Comparison

To effectively benchmark BioFigRank on the figure ranking task using the shared dataset, we compared our figure ranking model with the state-of-the-art listwise model, ListNet, as well as our previous unsupervised model. We also implemented a random model as a lower-bound.

- **Random:** For each article, we generated a randomized permutation of figures it contains, which was used as a ranking output. We repeated this process 100 times, and average the ranking performance to evaluate this random model.
- **Baseline:** This system duplicates the unsupervised algorithm in previous study [16].
- **ListNet:** We adapted the implementation in [47] for the figure ranking task. The learning rate is empirically set to 0.0009, and the iteration number is 200.
- **BioFigRank:** Extended the ListNet model above using a new loss function based on top 2 probability distributions; the learning rate was 0.0009 and the iteration number was 200.

## Results

### Performance Comparison among Different Systems

Table 1 shows the 10-fold cross-validation results for different systems. BioFigRank performed the best across all the metrics, yielding the best score of 0.808, 0.829 and 0.791, respectively for ∼WER-RK, NDCG and ∼WER-FR. BioFigRank outperformed ListNet and the differences are statistically significant based on 1-tail paired T-test. As expected, all three models outperformed the random model and both supervised approaches outperformed the unsupervised baseline.

**Table 1:** Performance of different systems.

Systems	∼WER-RK	NDCG	∼WER-FR
Random	0.649±0.087	0.748±0.080	0.649±0.106
Baseline	0.795±0.182	0.824±0.136	0.771±0.257
ListNet	0.804±0.179	0.825±0.143	0.788±0.248
BioFigRank	**0.808±0.178** **	**0.829±0.144** *	**0.791±0.248** **

(**p<0.05, *p<0.1).

**Figure 4:**
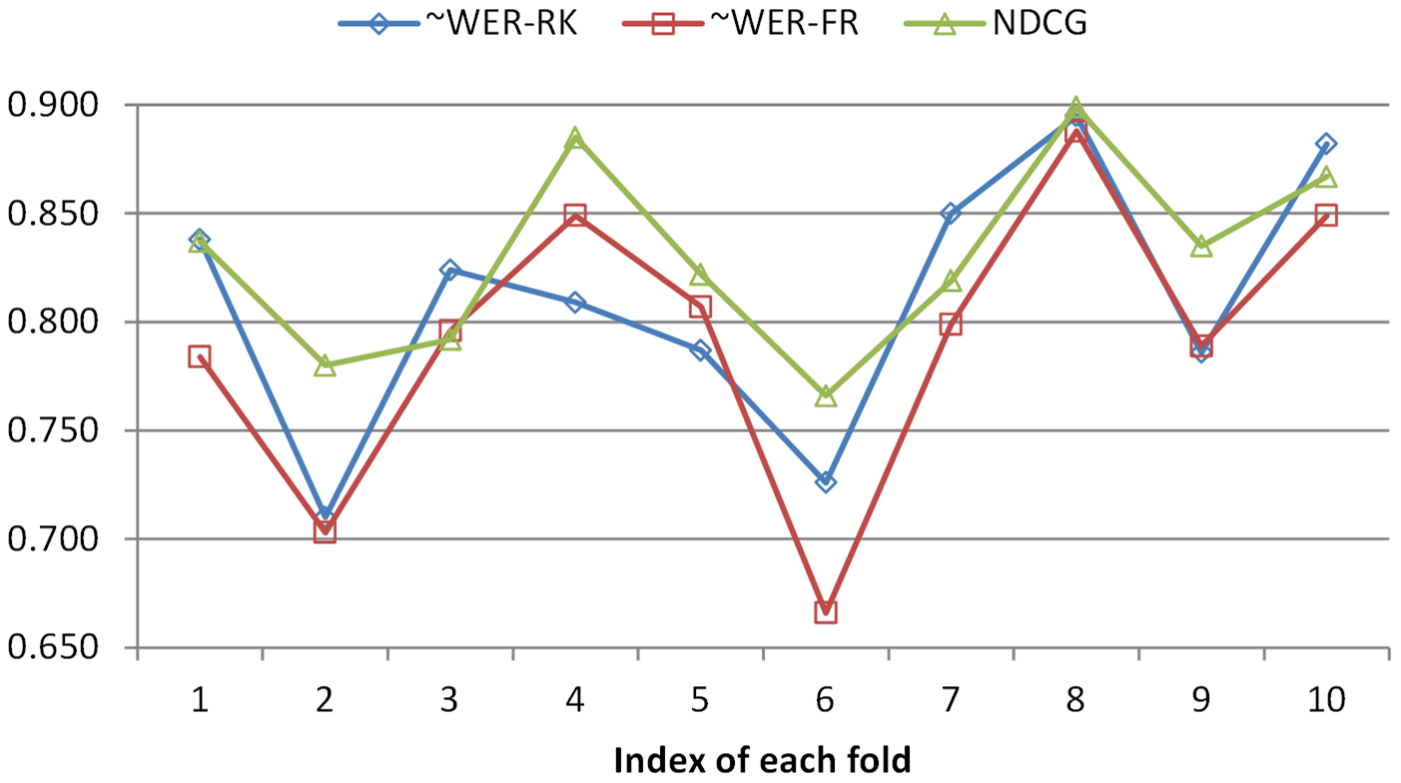
10-fold cross validation performance of Biofigrank.

As shown in Figure 4, we noticed that the ranking performance vary considerably among different folds, especially for ∼WER-RK ranging from 0.71 to 0.895 and ∼WER-FR from 0.666–0.888. NDCG shows relatively stable performance, but still shows the gap between 0.761 and 0.899. This indicates that the characteristics of ranking relationships among figures in different articles could be very different, as some articles may have different strategies to organize the figures and associated content.

### Analysis of System Performance based on Number of Figures per Article

**Figure 5:**
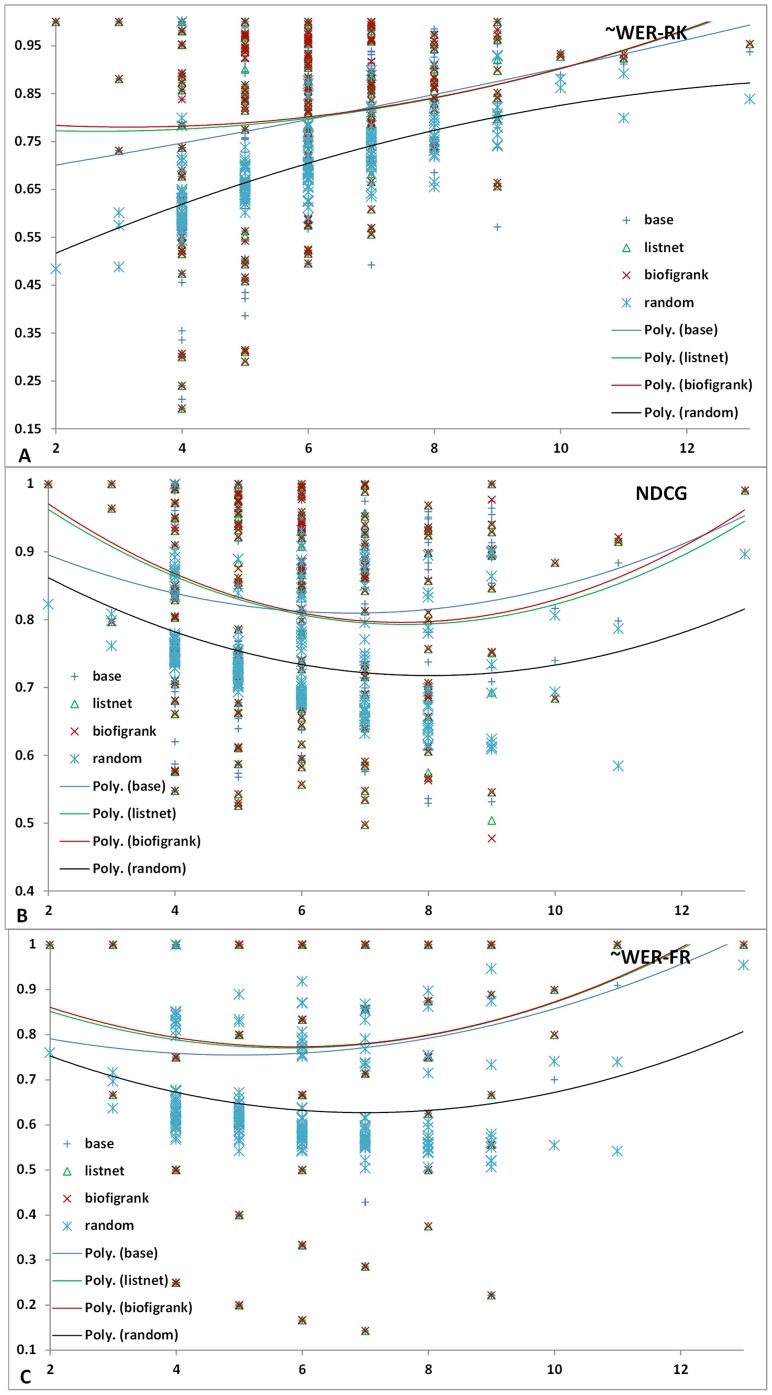
Scatter graph with regard to ranking performance and number of figures. A: ∼WER-RK metrics; B: NDCG metrics; C: ∼WER-FR metrics.

Figure 5 shows the scatter graphs with regard to the ranking performance and the number of figures. The trend lines are based on 2nd-order polynomial regression. As shown in Figure 5, all systems exhibit similar performance trend with number of figures. With NDCG (Figure 5B) as the evaluation metrics, Biofigrank slightly outperformed Listnet across different figure numbers. With two other metrics (∼WER-RK and ∼WER-FR) (Figure 5A and 5C), Biofigrank outperformed ListNet when the number of figures is below 6 and there is little noticeable difference in performance when the number of figures in each article is above 6.

**Figure 6:**
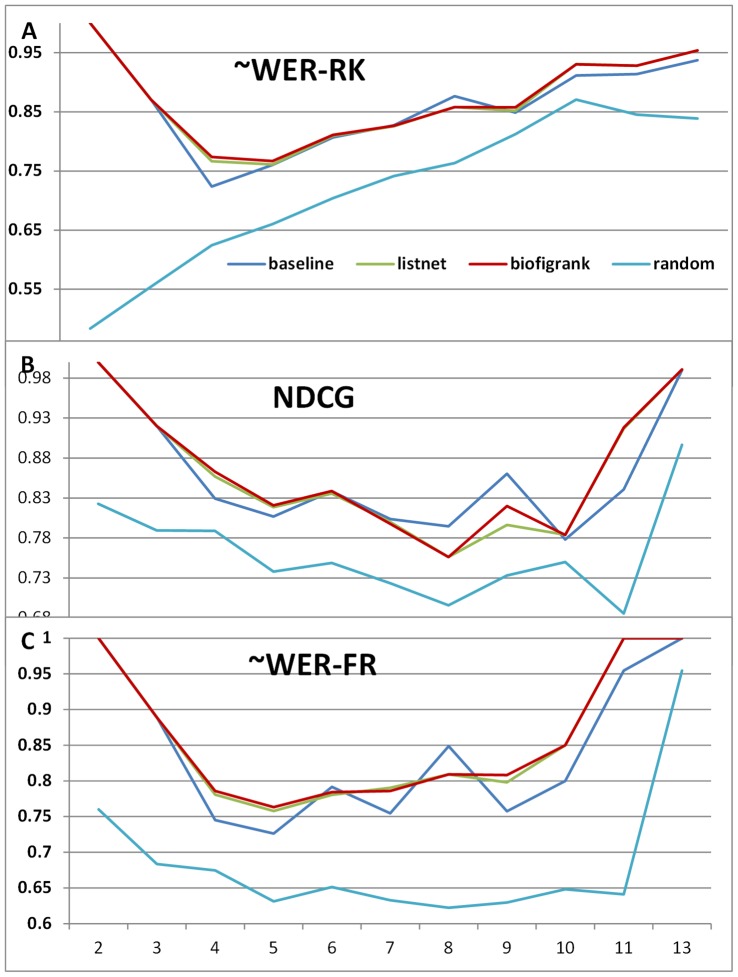
Ranking performance trend as the number of figures changes. A: ∼WER-RK metrics; B: NDCG metrics; C: ∼WER-FR metrics.

We observed a similar trend in Figure 6 which shows the performance curve with figure numbers where each data point is the average of ranking metrics over articles with certain number of figures. In addition, we found that at some point an unsupervised model can do a better job. For example, it achieved a noticeable peak at ∼WER-RK and ∼WER-FR (Figure 6A and 6C) with the figure number of 8, and one at NDCG(Figure 6B) with the figure number of 9. Based on 1-tail paired T-test, the advantage of unsupervised model on articles containing 8 figures in terms of ∼WER-RK and ∼WER-FR is not statistically significant, but the better performance at NDCG on articles containing 9 figures are statistically significant (*p*<0.001).

Note that in Figure 6A, the random model shows different trend at the starting point because when the figure number is 2, the ∼WER-RK performance of random model (either 0 or 1 for each random trial) would be around 50%, but all the other models got the correct ranking easily which gave the ∼WER-RK score of 1 at that point.The random system achieved another peak point at 13 in Figure 6C, because there are only 3 ranking groups for 13 figures in that article with many tie relationships (see details in the discussion), leading to the expectation of ∼WER-FR at 0.9527.

### Contributions of Different Feature Categories

To better understand how effective the four feature categories are, we analyzed their contributions in BioFigRank by removing one category at one time, as shown in Table 2. “Cent” indicates the centrality features, “Freq” for frequency features, “LDA” for LDA-based topic features, and “Struc” for structural features.

**Table 2:** Effectiveness of different feature categories.

Feature Categories	∼WER-RK	NDCG	∼WER-FR
All	**0.808±0.178**	0.829±0.144	0.791±0.248
All w/o Cent	0.791±0.19***	0.815±0.146***	0.775±0.251**
All w/o Freq	0.803±0.18	0.825±0.144	0.792±0.244
All w/o LDA	0.810±0.169	**0.833±0.137**	0.786±0.252
All w/o Struc	0.806±0.181	0.83±0.144	**0.793±0.144**

(***p<0.01, **p<0.05).

We can see that the centrality features play the most important role, as removing the features leads to the worst performance of 0.791/0.815/0.775 (see row 3). This suggests the centrality-based features are a good indicator of figures being the central point of the current article.

Mixed trends are found for the other three categories. The LDA features are shown to be helpful to identify the most important figures with the ∼WER-FR increasing from 0.786 to 0.791, but slightly hurt the performance on the overall ranking, decreasing the ∼WER-RK/NDCG value from 0.810/0.833 to 0.808/0.829 respectively (compare row 1 and row 5 in Table 2). In contrast, frequency features seem to help the overall ranking, increasing the ∼WER-RK/NDCG from 0.803/0.825 to 0.808/0.829, respectively, but without affecting much on the performance of identifying the most figures (0.792 vs. 0.791). Structural features do not seem to contribute much to the ranking performance, as removing them results in only marginal performance differences.

### Results of Forward Greedy Feature Selection (FGFS)

**Figure 7:**
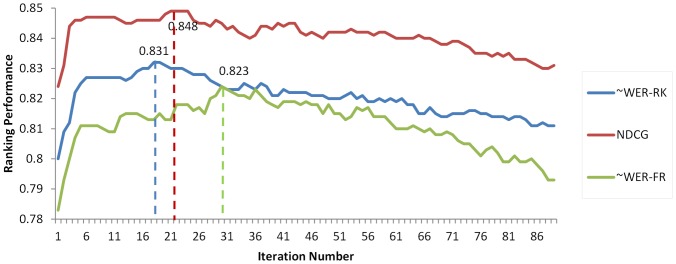
Performance curve with each feature selection iteration.

We conducted forward feature selection based on three metrics as shown in Figure 7. It shows that the performance of BioFigRank increases while incrementally adding the optimal feature at each iteration, but after a number of iterations the performance curve turns flat or even goes slightly down. FGFS achieved the peak performance with 18 optimal features using the metrics of ∼WER-RK, reached the peak performance with 21 optimal features using NDCG, and yielded the best score with 30 optimal features using ∼WER-FR.

Results of Biofigrank using different feature selection strategies are presented in Table 3, where “Indiv_X” selected the same number (shown in parentheses) of top features as FGFS, only based on the performance metric “X” when using each individual feature, “FGFS_Combined” combined the features selected by three FGFS based feature sets in terms of three metrics as shown in Figure 7.

**Table 3:** Ranking performance using different feature selection methods.

Feature Selection Methods	∼WER-RK	NDCG	∼WER-FR
W/O FS(89)	0.808±0.178	0.829±0.144	0.791±0.248
FGFS_∼WER-RK(18)	**0.831±0.172** ***	0.847±0.138***	0.803±0.258
Indiv_∼WER-RK(18)	0.805±0.177	0.827±0.139	0.775±0.258
FGFS_NDCG(21)	0.827±0.166**	**0.848±0.133** ***	0.807±0.245
Indiv_NDCG(21)	0.808±0.180	0.830±0.141	0.783±0.254
FGFS_∼WER-FR(30)	0.820±0.172**	0.839±0.141*	**0.823±0.227** ***
Indiv_∼WER-FR(30)	0.801±0.185	0.823±0.145	0.785±0.248
FGFS_Combined(35)	0.812±0.179	0.832±0.143	0.792±0.250

(***p<0.01, **p<0.05, *p<0.1).

The results demonstrate the high effectiveness of FGFS based method compared with the naïve method pooling together top-performed features (FGFS vs. Indiv in Table 3). Most “Indiv_X” methods either can't even improve the performance or neglectable improvement. In contrast, FGFS based method can significantly improve the ranking performance across all three metrics, achieving the best ∼WER-RK of 0.831, NDCG of 0.848, and ∼WER-FR of 0.823, compared with the original BioFigRank performance of 0.808, 0.829 and 0.791 respectively.

### Learning Curve and Descriptive Statistics Analysis

**Figure 8:**
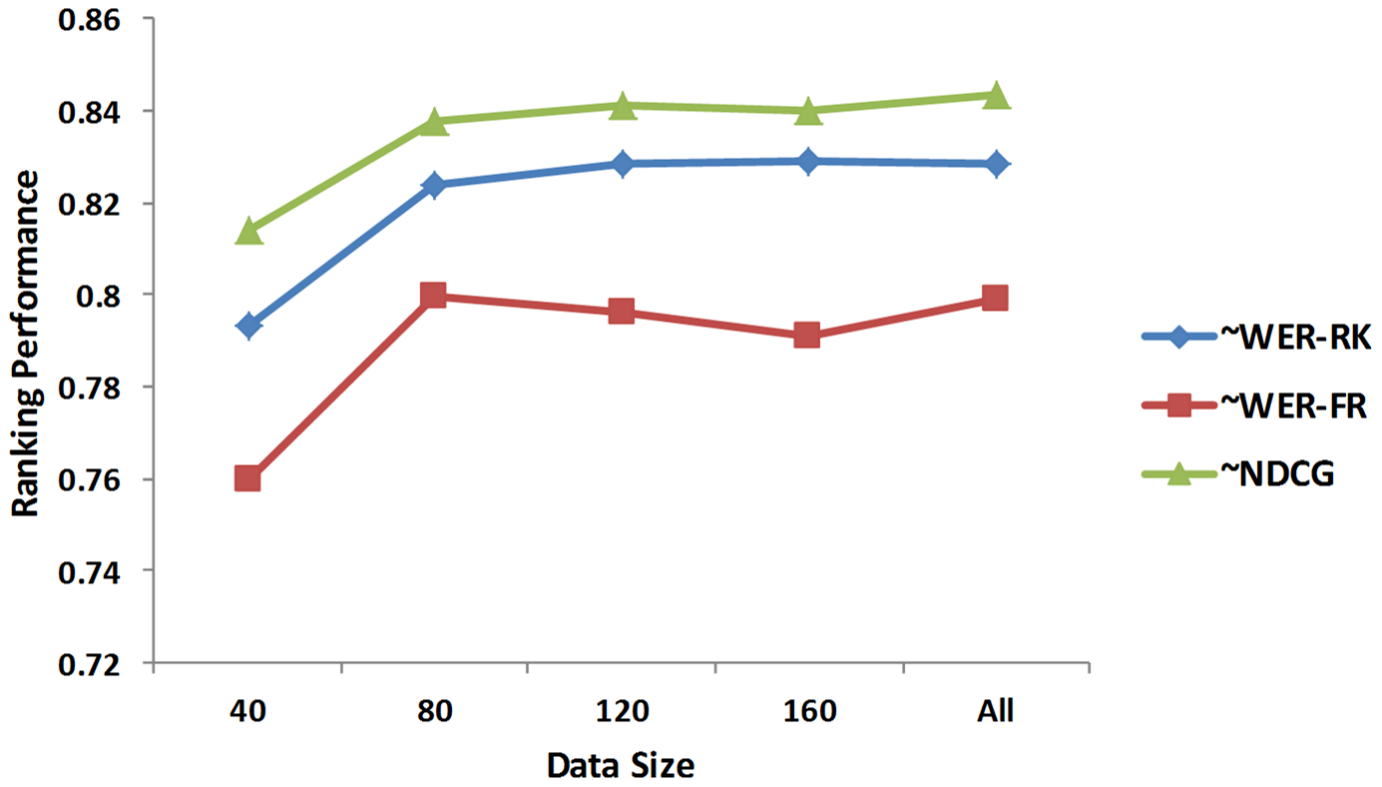
Learning curve of BioFigRank.

We analyzed the learning curve of BioFigRank and descriptive statistics on its performance with the feature selection setting of FGFS_∼WER-RK in Table 3. Trends using other FGFS methods are similar. We randomly shuffled all the articles 5 times, and from each shuffled list we incrementally selected 40, 80, 120, 160 and all articles to do the 10-fold cross validation, finally the average of cross validation results over 5 times is calculated at 40, 80, 120, 160 and all articles. The resulting learning curve is shown in Figure 8.

We observed the typical trend of increasing learning performance with more data, although there are some fluctuations at some specific point, e.g. when the data size increases from 120 to 160, the ∼WER-FR performance drops a little bit. The fact that the performance curve line rises quickly at the beginning and gently at the end illustrates the data we used for our experiments is in a good size.

**Figure 9:**
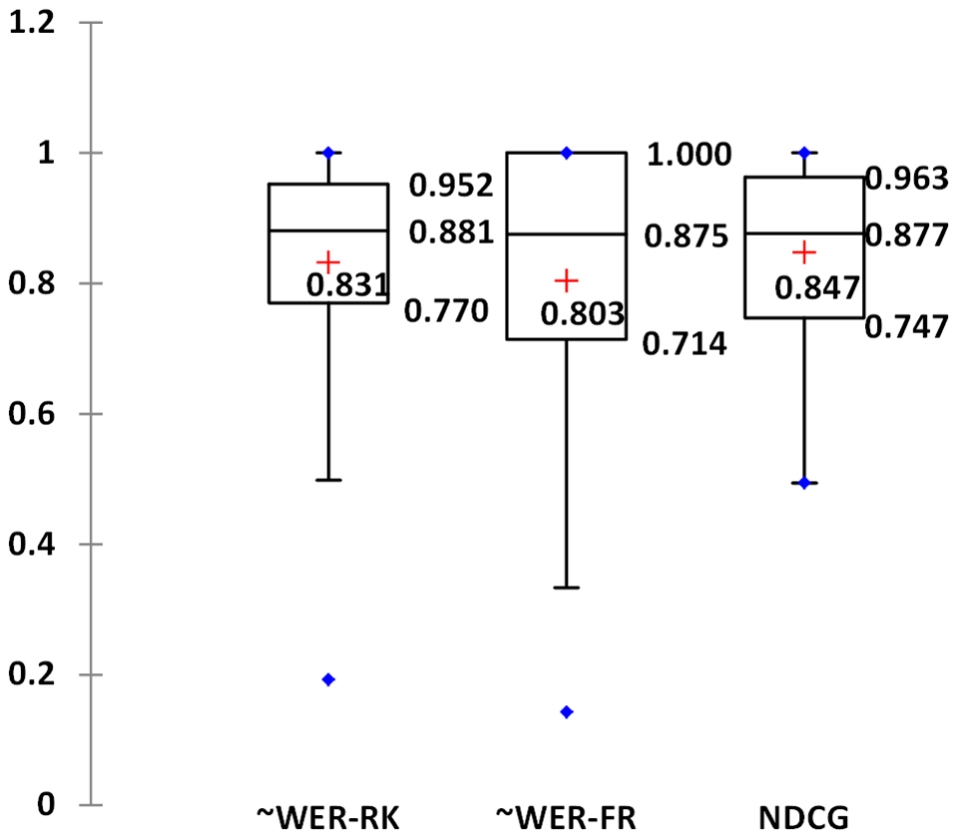
Box plots of BioFigRank performance in three metrics.

Figure 9 shows the descriptive statistics of the BioFigRank's 10-fold cross validation performance when using all the data with feature selection, where “+” indicates the mean value in Table 3. We see that all the boxes shifted to the high end, which shows a negative skewedness on the normality of the sample's distribution. The median score of three performance measurements are all around 0.88, demonstrating that BioFigRank can achieve the performance equal to or larger than 0.88 on 50% of the articles. The inter-quartile range represents the middle 50% of the articles, which is 0.77–0.952 for ∼WER-RK, 0.747–0.963 for NDCG and 0.714–1.000 for ∼WER-FR. The upper whisker of ∼WER-FR overlapped with the 3^rd^ quartile of 1.000, suggesting that about 25% of the articles got the perfect score of 1 on recognizing the most important figures.

### Comparative Experiments on Machine Computation versus Human Intelligence

In this experiment, we further evaluated how well BioFigRank performs compared to human subjects with different levels of in-domain knowledge. Three levels were considered in this study:

1. First Author: Biologist who is the first author of an article but not the corresponding author (who produced the gold standard annotation) at the same time.
2. Non-Author-In-Domain-Expert: Biologist who is not the author or co-author of an article but who works in the same field of the corresponding author of the article.
3. Non-Author-Out-Domain-Expert: Biologist who is not the author or co-author of an article and who may or may not work in the same field of the corresponding author of the article.

We used the same dataset of 202 biological articles for this experiment. First we manually checked the contact information of the first author of each paper who is not the corresponding author. Out of 202 articles, 97 non-corresponding first authors can be reached and asked to rank figures in their published articles. 27 of them responded, resulting in a data set with annotations from both first authors and corresponding authors. We run BioFigRank (with feature selection setting of FGFS_∼WER-RK in Table 3) on this data to compare the performance of first authors and BioFigRank against the gold standard annotation by corresponding authors as in Table 4.

**Table 4:** BioFigRank compared with first authors.

	∼WER-RK	NDCG	∼WER-FR
BioFigRank	0.827±0.185	0.845±0.137	0.808±0.261
First Author	**0.924±0.145**	**0.929±0.129**	**0.896±0.239**
T-test p value	0.017**	0.013**	0.107

(**p<0.05).

We can see, not surprisingly, the first author achieved significantly better performance with ∼WER-RK of 0.924, NDCG of 0.929 and ∼WER-FR of 0.896, compared with BioFigRank (0.827, 0.845 and 0.808). It also shows that getting the very first rank correct (∼WER-FR of 0.808) is more challenging than getting a better overall ranking with only minor local inconsistencies (∼WER-RK of 0.827, NDCG of 0.845).

For the second comparison, we asked some biologists to rank figures for non-authored biological articles that are of their own interests. For the collected 63 articles from 5 biologists, we asked corresponding authors to rank figures in their published articles to get the gold standard annotation as we did before. 16 authors responded and then we run BioFigRank to compare its performance with Non-Author-In-Domain-Expert biologists, as shown in Table 5.

**Table 5:** BioFigRank compared with Non-Author-In-Domain-Expert biologists.

	∼WER-RK	NDCG	∼WER-FR
BioFigRank	**0.822±0.112**	0.846±0.089	**0.789±0.214**
Non-author in-domain-expert biologist	0.805±0.162	**0.857±0.133**	0.768±0.336
T-test p value	0.341	0.352	0.405

We notice that BioFigRank can achieve better performance in terms of ∼WER-RK and ∼WER-FR (0.822 vs. 0.805; 0.789 vs. 0.768), but slightly worse in terms of NDCG(0.846 vs. 0.857). Although those are not statistically significant, BioFigRank demonstrate that it can perform as well as Non-Author-In-Domain-Expert biologists on the figure ranking task, offering human-level intelligence to derive structural relations among figures.

Finally, we randomly selected 44 articles from the 202 data set. We recruited 6 biologists and ask them to rank figures for those articles. Similarly, we run BioFigRank on the 44 articles to compare its performance with Non-author-Out-Domain-Expert biologists, as shown in Table 6.

**Table 6:** BioFigRank compared with Non-author-Out-Domain-Expert biologists.

	∼WER-RK	NDCG	∼WER-FR
BioFigRank	**0.822±0.178**	**0.837±0.151**	**0.807±0.250**
Non-author out-domain-expert biologist	0.721±0.232	0.802±0.211	0.671±0.299
T-test p value	0.016**	0.190	0.019**

(**p<0.05).

The results show that BioFigRank can significantly (p<0.05) outperform Non-author-Out-Domain-Expert biologists in both ∼WER-RK (0.822 vs. 0.721) and ∼WER-FR(0.807 vs. 0.671). For NDCG, BioFigRank achieved 4.4% gain (0.837 vs. 0.802) over the non-author-out-domain-experts although it is not statistically significant.

## Discussion

Figure ranking is drawing more and more attention in the biological research community and developing computational models to provide effective solutions is extremely important. We developed a new ranking model, BioFigRank, which implemented an extended loss function in the listwise learning-to-rank framework. Although the main difference between BioFigRank and ListNet lies in the loss functions based on top one versus top two permutation probability, we have explored and implemented a different model inference for the adapted model , as shown in Eq. (7). Our comparative analysis demonstrates that the computational model of BioFigRank can provide human intelligence, at the Non-Author expert biologist level, on deriving structural relations among figures in a biological article. This will open up a lot of opportunities to integrate this intelligence in facilitating biologists' information seeking needs at this big data era.

Our experimental results demonstrate that BioFigRank outperforms our previous unsupervised model and one of the state-of-the-art listwise learning-to-rank models: ListNet. It validates our hypothesis that the top 2 permutation probability can better capture the difference between the system ranking and ground truth ranking, leading to more robust optimization in the learning process. In particular, BioFigRank performs very well when the number of articles ranges from 4 to 6 as shown in Figure 6, while Figure 2 also shows that larger number of articles contain 4–6 figures. That explains that the BioFigRank model can achieve the best performance on the figure ranking task. More extensive evaluations will be conducted in the future, with the goal of further validating how much the advantages of BioFigRank model over other models can be translated in improving other practical applications, such as figure search and information retrieval.

As shown in Figure 3, BioFigRank performs considerably different on different sets of articles. On the other hand, different models also perform differently on different articles. From Figure 6 we can see, unsupervised model can achieve even better performance than supervised models on some subset of articles, e.g. articles with 9 figures with respect to the NDCG metric. The number of figures is one factor, and it suggests that BioFigRank might need to be personalized so that articles with different characteristics can be treated differently, leading to further boosted performance. A classification framework could be integrated to classify articles into different clusters where different figure ranking strategies could be applied in favor of this specific cluster.

Our analysis shows that the figure ranking performance has an overall trend of favoring articles with larger number of figures in terms of ∼WER-RK as in Figure 5A, which seems count-intuitive. In reality, if we assume all the permutations occur randomly with the same probability, the expectation value of ∼WER-RK will increase with the larger number of figures in each article. For example, the expectation value (assuming each permutation has a same probability) of ∼WER-RK is 0.5 on 2 figures, 0.537 for 3 figures, 0.596 for 4 figures, and 0.643 for 5 figures.

**Figure 10:**
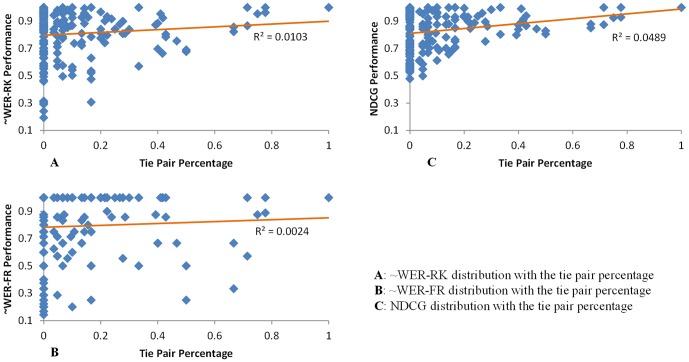
Impacts of tie relationship on the figure ranking performance. Scatter graph between tie pair percentage of an article and the figure ranking performance of BioFigRank in three metrics.

Another factor related to favoring larger number of figures is that in our figure ranking task, we allow tie relationships in the gold standard annotation. For articles with larger number of figures, there is a lager possibility to be assigned tie relationship among them. We found that the average number of figures on articles containing at least one tie relation is 6.538, larger than the average of 5.369 on articles containing no tie relations. Because of the existence of tie-relations, the performance expectation of random assignment increases when the number of figures increases. As shown in Figure 6B and 6C, there are irregular peak points for NDCG and ∼WER-FR when the figure number is 11 or 13. The phenomenon is due to the fact that only one article in the dataset incorporates 13 figures containing many tie relations and hence a very high performance score was achieved. The scatter graph in Figure 10 shows how tie pairs relate to the BioFigRank's performance. It indicates that the ranking performance tends to increase with a higher tie pair percentage, while the impact on recognizing the most important figure (Figure 10B) seems less than ranking all the figures (Figure 10A&C).

Different feature categories contribute differently to the ranking system. We saw that LDA and frequency features contribute differently in terms of different performance metrics, possibly because the LDA features are generated using top 4 topics based on their relevancy to abstract or title and thus might be good for identifying most important figures. Although intuitively the figure with higher frequency is expected to be more important, this is frequently untrue in reality, especially for recognizing the most important figure. Therefore our results show that frequency features are more helpful on the overall ranking of the figures than the identification of the most important figure. Our results also show that structural features contribute little. There are two possibilities: the task of sub-figure extraction introduces noise, and link features might be redundant with centrality features.

Another interesting finding is that individual features, through FGFS feature selection, can interact implicitly with each other for better performance. We found that simply selecting and pooling together best-performing individual features is not as effective as forward greedy feature selection method for overall system performance. The results demonstrate the effective of iterative FGFS method for optimal feature selection.

The top 5 features selected by FGFS are:

1. The similarity between the figure's associated context and the abstract of the article;
2. The frequency of the figure referred in the Results section of the article;
3. The similarity between the figure caption and Results section of the article;
4. The accumulative similarity between the figure's associated context and the top 2 LDA topics based on relevancy to abstract;
5. The similarity between the figure caption and the abstract of the article.

It shows that a figure's associated context is complementary to its caption as we presented in the Results section. Latent Dirichlet allocation (LDA) based topic analysis offers another effective method of slicing and sifting important information for the figure ranking task. As stated earlier, the LDA feature category does not appear to improve the performance of BioFigRank. However, through FGFS feature selection, one of the individual LDA features was ranked within top 4 features. It suggests that not all the features from each category contribute positively, and individual features from different categories can interact each other implicitly in a different way to improve the system performance.

In this study, we didn't explicitly measure the inter-personal annotation agreement due to the challenge of our task. But the results in Table 3 demonstrated a good agreement rate between first author and corresponding authors.

## Conclusions

We investigated learning-to-rank approaches for figure ranking, in which figures appearing in the same biomedical article are ranked and evaluated against corresponding authors' annotation. We implemented a new computational figure ranking model, BioFigRank, which further extended the loss function in ListNet, a state-of-the-art listwise learning-to-rank model. Experimental results show that BioFigRank outperforms the competitive ListNet model as well as the unsupervised baseline model. We further applied forward greedy feature selection (FSFS), and the ranking performance of BioFigRank is significantly improved, achieving the best ∼WER-RK of 0.831, NDCG of 0.848, and ∼WER-FR of 0.823. Compared with human experts, BioFigRank performs as well as non-author-in-domain-expert biologists and better than non-author-out-domain-expert biologists.

Looking ahead, we plan to explore approaches to further enhance BioFigRank's performance. For example, we will select salient features by exploring a more systematic way such as dynamic programming based feature selection. Since we found BioFigRank's performance is article-dependent, article centric approaches will be a future direction. In addition, we will integrate figure ranking into other applications, such as document retrieval, biocuration, and literature-based approaches for assisting high-throughput data analyses and interpretation.
